# *ROS1* mutations promote an immunosuppressive tumor microenvironment via *MYC* to confer immune evasion in head and neck cancer

**DOI:** 10.20517/cdr.2025.124

**Published:** 2025-08-22

**Authors:** Chao Fang, Qin Zhang, Rui Fang, Ying Li, Jing Bai, Xiaojing Huang, Jingting Lu, Dongsheng Chen, Yanxiang Zhang, Zuhong Chen

**Affiliations:** ^1^The School of Clinical Medicine, Fujian Medical University, Fuzhou 350000, Fujian, China.; ^2^Department of Otolaryngology-Head & Neck Surgery, The First Hospital of Putian, Putian 351100, Fujian, China.; ^3^State Key Laboratory of Neurology and Oncology Drug Development, Jiangsu Simcere Diagnostics Co., Ltd., Nanjing 210018, Jiangsu, China.‌; ^#^Authors contributed equally.

**Keywords:** Head and neck cancer, ICI resistance, *ROS1* mutation, tumor microenvironment

## Abstract

**Aim:** Immune checkpoint inhibitors (ICIs) have transformed cancer therapy; however, their efficacy in head and neck cancer (HNC) remains limited, with only a minority of patients achieving durable responses. Understanding the molecular mechanisms underlying ICI resistance in HNC is therefore crucial.

**Methods:** We conducted an integrative analysis of genomic, transcriptomic, and clinical data from 139 ICI-treated HNC patients (MSKCC cohort) and 502 treatment-naïve HNC cases (TCGA cohort). *ROS1* mutation status, tumor mutational burden (TMB), neoantigen load, immune cell infiltration (via CIBERSORT), and immune-related gene expression were evaluated. Gene set enrichment analysis (GSEA) was performed to identify dysregulated pathways. Survival outcomes were assessed using Kaplan-Meier analysis and Cox regression, with statistical significance defined as *P* < 0.05.

**Results:** Patients harboring *ROS1* mutations exhibited significantly poorer outcomes following ICI therapy, with shorter median overall survival [OS: 5.0 *vs.* 11.0 months, hazard ratio (HR) = 3.22, 95%CI: 1.26-8.19, *P* = 0.011] compared to *ROS1* wild-type counterparts. Multivariate analysis confirmed *ROS1* mutation as an independent predictor of poor OS in ICI-treated patients (HR = 4.78, 95%CI: 1.70-13.43, *P* = 0.003). In contrast, *ROS1* mutations showed no prognostic significance in the treatment-naïve TCGA-HNC cohort (*P* = 0.26), confirming their role as a predictive (not prognostic) biomarker for ICI response. Interestingly, despite exhibiting higher TMB and neoantigen levels, *ROS1*-mutant patients showed inferior survival, underscoring the context-dependent limitations of TMB as a predictive biomarker. Mechanistically, *ROS1*-mutant tumors displayed an immunosuppressive tumor microenvironment characterized by diminished CD8^+^ T cell infiltration, attenuated interferon-γ signaling, and downregulation of immune-related genes (*CXCL9*, *CXCL10*, *IFNG*, *PD-L1*). GSEA revealed enrichment of *MYC* pathway activity in *ROS1*-mutant tumors, which suppressed antigen presentation and T cell activation pathways.

**Conclusion:** *ROS1* mutations drive ICI resistance in HNC by promoting an immunosuppressive TME via *MYC*-mediated transcriptional reprogramming, impairing antigen presentation and T cell function. Incorporating *ROS1* status into biomarker panels may improve patient stratification and guide combinatorial therapies targeting both immune evasion and oncogenic pathways.

## INTRODUCTION

Head and neck cancer (HNC), encompassing malignancies of the oral cavity, pharynx, and larynx, is the sixth most common cancer globally, with approximately 890,000 new cases and 450,000 deaths reported in 2022^[1]^. Despite advances in multimodal therapies, the prognosis for advanced HNC remains poor, with a 5-year survival rate below 50% for recurrent or metastatic disease^[1,2]^.

Treatment options for recurrent or metastatic HNC have evolved significantly over the past decade, improving survival outcomes^[3,4]^. The immune checkpoint inhibitors (ICIs) pembrolizumab and nivolumab are FDA-approved for cisplatin-refractory recurrent or metastatic HNC. Current national and regional guidelines recommend first-line therapy based primarily on programmed cell death ligand 1 (PD-L1) expression levels. Options include pembrolizumab (with or without chemotherapy) or cetuximab-based regimens (e.g., cetuximab combined with platinum/5-FU chemotherapy)^[5-7]^. For second-line treatment, nivolumab or pembrolizumab is advised, with alternatives including cetuximab (with or without chemotherapy) or biomarker-directed therapy. Despite these advances, sustained clinical benefits are observed in only 10%-20% of patients, highlighting the urgent need for predictive biomarkers to optimize patient stratification^[8,9]^.

Recent studies have identified tumor mutational burden (TMB), microsatellite instability (MSI), and PD-L1 expression as key determinants of ICI response; however, their predictive power in HNC remains suboptimal. For instance, PD-L1 positivity is associated with improved ICI response in HNC, yet approximately 80% of PD-L1-positive patients fail to achieve objective responses^[7]^, highlighting the complexity of tumor-immune interactions^[10]^. Similarly, while high TMB correlates with enhanced neoantigen presentation and ICI efficacy in melanoma and lung cancer^[11,12]^, its utility in HNC is limited by molecular heterogeneity^[5,8]^. These observations emphasize the need for comprehensive molecular characterization and immune profiling to integrate prognostic and predictive biomarkers into clinical practice.

ROS1, a receptor tyrosine kinase, is implicated in the carcinogenesis of multiple cancers. *ROS1* fusions are established therapeutic targets in lung adenocarcinoma, with inhibitors such as entrectinib and taletrectinib demonstrating efficacy^[13-15]^. However, the role of somatic *ROS1* mutations (*ROS1*-Mut) in HNC remains unexplored. Emerging evidence suggests that somatic mutations in driver genes can modulate tumor immunogenicity; for example, *ALK* rearrangements, *EGFR* mutations, and *KRAS* mutations correlate with immunosuppressive tumor microenvironments (TMEs)^[16,17]^. Paradoxically, colorectal cancers with an ultramutated phenotype exhibit significantly higher objective response rates and more favorable outcomes following ICI treatment compared to dMMR/MSI-H tumors^[18]^. These findings raise the intriguing possibility that *ROS1*-Mut may similarly influence immune response in HNC.

Given the critical need to overcome ICI resistance in HNC, we hypothesize that *ROS1*-Mut might drive immunosuppressive mechanisms similar to those induced by oncogenic drivers such as *ALK* or *EGFR*. To investigate this, we integrated multi-omics analyses of 139 ICI-treated HNC patients (MSKCC cohort) and 502 treatment-naïve cases (TCGA cohort) with three specific aims: (1) determine whether ROS1-Mut predict poor ICI response independently of TMB/PD-L1; (2) characterize the immunogenomic landscape of *ROS1*-Mut tumors; and (3) elucidate mechanistic links between *ROS1*-Mut and *MYC*-driven immune evasion. This study identifies *ROS1*-Mut as candidate mediators of ICI resistance and proposes potential therapeutic strategies.

## METHODS

### Study cohorts and data acquisition

We analyzed two independent HNC cohorts: 139 advanced HNC patients treated with ICIs [anti-programmed death 1 (PD-1)/PD-L1 ± anti-cytotoxic T-lymphocyte-associated protein 4 (CTLA-4)] from the MSKCC cohort, and 502 treatment-naïve HNC cases from The Cancer Genome Atlas (TCGA) cohort. Clinical and genomic data were retrieved from the following sources: (1) MSKCC cohort from Samstein *et al.*^[9]^; (2) whole-exome sequencing (WES) and TMB data from Hoadley *et al.*^[19]^; (3) RNA-seq data from the Genomic Data Commons (GDC; https://portal.gdc.cancer.gov/); and (4) survival data from the UCSC Xena Browser (https://xenabrowser.net).

### Genomic profiling

*ROS1* mutation was defined as non-synonymous somatic mutations, including missense, nonsense, splice-site mutations, and in-frame indels in the coding region of the *ROS1* gene. TMB was defined as the total count of non-synonymous mutations per megabase (mut/Mb), with a TMB-high (TMB-H) threshold set at > 10 mut/Mb. Neoantigen prediction was performed as previously described^[20]^. Expressed somatic variants and patient-specific HLA alleles (predicted using POLYSOLVER) were used as inputs for the NetMHCpan 4.0 algorithm^[21]^. Strong-binding peptides (IC50 < 500 nM) were counted as neoantigens to calculate tumor neoantigen burden (TNB).

### Transcriptomic and immune analyses

Immune cell composition was estimated using CIBERSORT, which quantified 22 immune cell subsets from TCGA RNA-seq data based on the LM22 signature matrix (1,000 permutations)^[22]^. Differential gene expression was analyzed using the R package DESeq2^[23]^, with thresholds of FDR < 0.05 and log_2_ fold change > 0.5. Immune-related genes were obtained from Danaher *et al.* to compare expression between *ROS1*-Mut and *ROS1*-wild-type (*ROS1*-WT) HNC cases in TCGA^[24]^. Gene set enrichment analysis (GSEA) was performed using the R Package ClusterProfiler v3.18.1^[25]^. Gene sets were considered significantly enriched at an adjusted *P* value < 0.05 (Benjamini-Hochberg correction).

### Statistical analysis

All statistical analyses were performed in R version 4.0.3 (http://www.r-project.org). Categorical variables were compared using Fisher’s exact test, and continuous variables were assessed with the Wilcoxon rank-sum test. Overall survival (OS) differences were evaluated using Kaplan-Meier curves (log-rank test) and Cox proportional hazards models. Multivariate analyses were adjusted for age, gender, metastatic status, TMB, and treatment regimen. PD-L1 expression across *ROS1* subgroups was compared using Fisher’s exact test, and the association between *ROS1* status and TMB was assessed using the Mann-Whitney U-test. Statistical significance was defined as two-sided *P* < 0.05.

## RESULTS

### Clinicopathologic characteristics of ICI-treated HNC patients

A total of 139 patients with advanced HNC were enrolled (median age 62 years; range, 17-81 years; 78.4% male). Patients were classified according to primary tumor site: oropharynx squamous cell carcinoma (37, 26.6%), oral cavity squamous cell carcinoma (25, 18.0%), larynx squamous cell carcinoma (9, 6.5%), nasopharyngeal carcinoma (9, 6.5%), sinonasal squamous cell carcinoma (6, 4.3%), hypopharynx squamous cell carcinoma (5, 3.6%), and unspecified head and neck squamous carcinoma (48, 34.5%).

Regarding ICI treatment, 131 patients (94.2%) received PD-1/PD-L1 inhibitor monotherapy, while 8 patients (5.8%) received combination therapy with CTLA-4 inhibitors. Tumor samples included 42 primary tumors (30.2%) and 97 metastatic lesions (69.8%), all analyzed by DNA-based next-generation sequencing (NGS). Detailed clinicopathologic characteristics are listed in Supplementary Table 1.

### *ROS1* mutations in MSKCC and TCGA HNC cohorts

**Figure 1:**
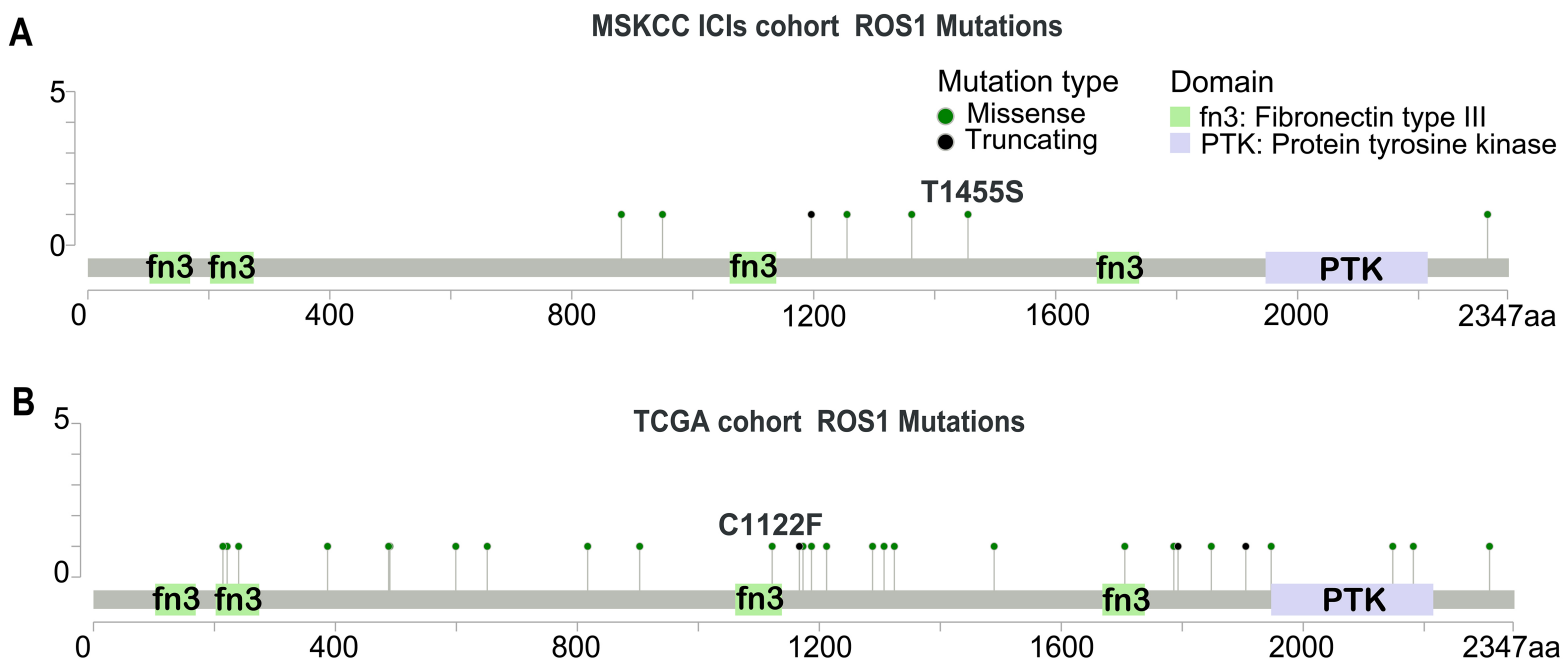
Somatic mutations of *ROS1* in patients with HNC. Lollipop plots show somatic mutation resulting in amino acid changes in (A) MSKCC HNC cohort and (B) TCGA HNC cohort. HNC: Head and neck cancer; TCGA: The Cancer Genome Atlas.

*ROS1*-Mut were detected in 5.0% (7/139) of the MSKCC cohort and 7.2% (36/502) of the TCGA-HNC cohort. Unlike *ROS1* kinase domain mutations that drive tyrosine kinase inhibitor (TKI) resistance in fusion-positive cancers^[26]^, somatic *ROS1*-Mut in both the MSKCC [Figure 1A] and TCGA [Figure 1B] cohorts were distributed broadly across the protein (e.g., extracellular, transmembrane, cytoplasmic, and kinase domains). Most variants were classified as variants of unknown significance (VUS) according to current CAP/AMP guidelines, and their clinical relevance requires functional validation.

### *ROS1* mutations are associated with poor survival in the ICI-treated HNC cohort

**Figure 2:**
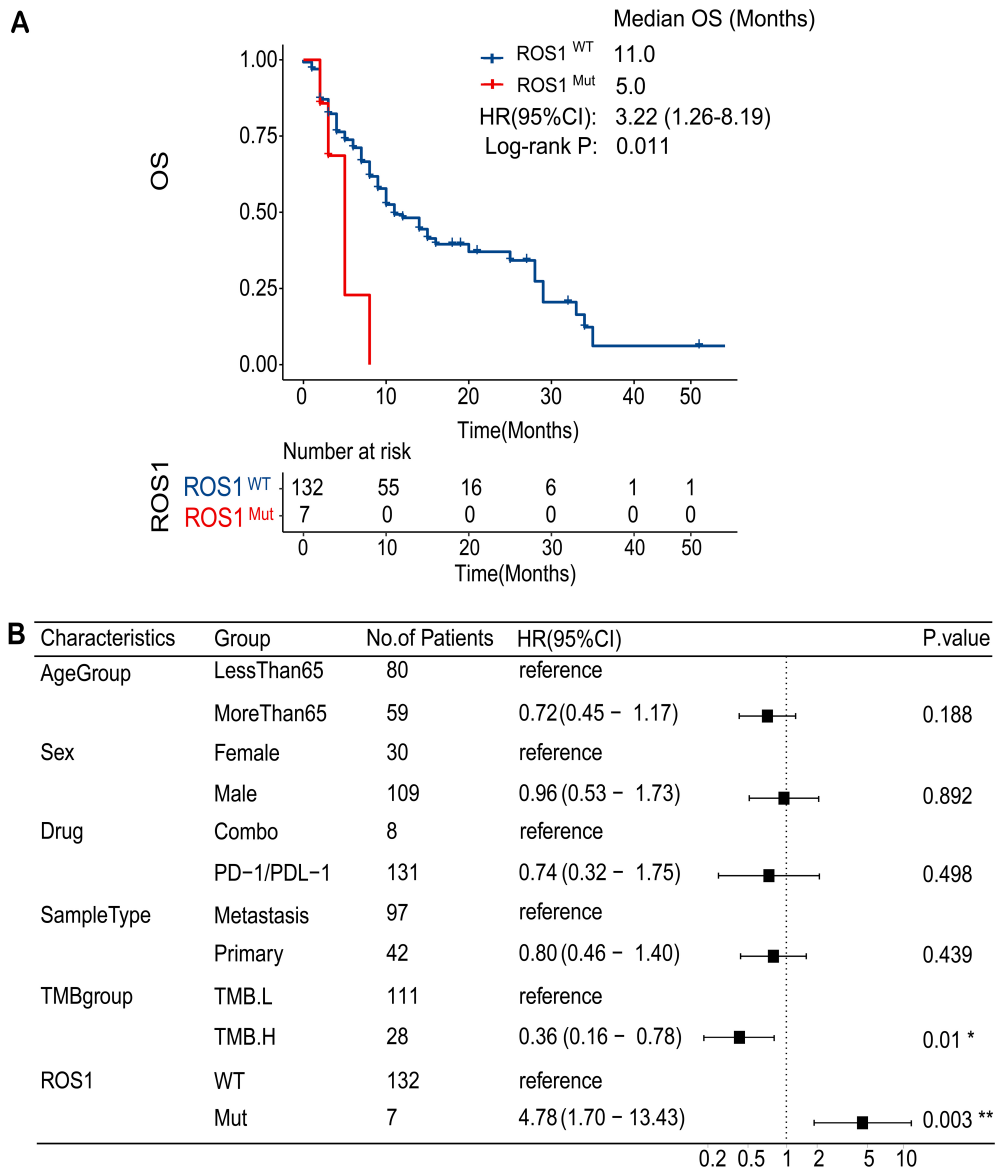
*ROS1* mutation as a predictor of ICI resistance in patients with HNC. (A) Kaplan-Meier survival curves comparing OS between *ROS1*-Mut and *ROS1*-WT groups in the ICI-treated HNC cohort; (B) Multivariate analysis of OS in the ICI-treated HNC cohort. ICI: Immune checkpoint inhibitor; HNC: head and neck cancer; OS: overall survival; *ROS1*-Mut: *ROS1* mutations; *ROS1*-WT: *ROS1*-wild-type.

To assess the impact of *ROS1*-Mut on treatment outcomes, we examined their association with response to ICI therapy. Patients with *ROS1*-WT mutations exhibited a median OS of 11.0 months, whereas patients with *ROS1*-Mut had a significantly shorter median OS of 5.0 months [Figure 2A]. The hazard ratio (HR) for OS comparing *ROS1*-Mut to *ROS1*-WT was 3.22 (95%CI: 1.26-8.19; *P* = 0.011). Multivariate analysis confirmed *ROS1* mutations as an independent predictor of poor OS in ICI-treated HNC patients (HR = 4.78; 95%CI: 1.70-13.43; *P* = 0.003), after adjusting for age, gender, metastatic status, TMB, and treatment regimen [Figure 2B].

**Figure 3:**
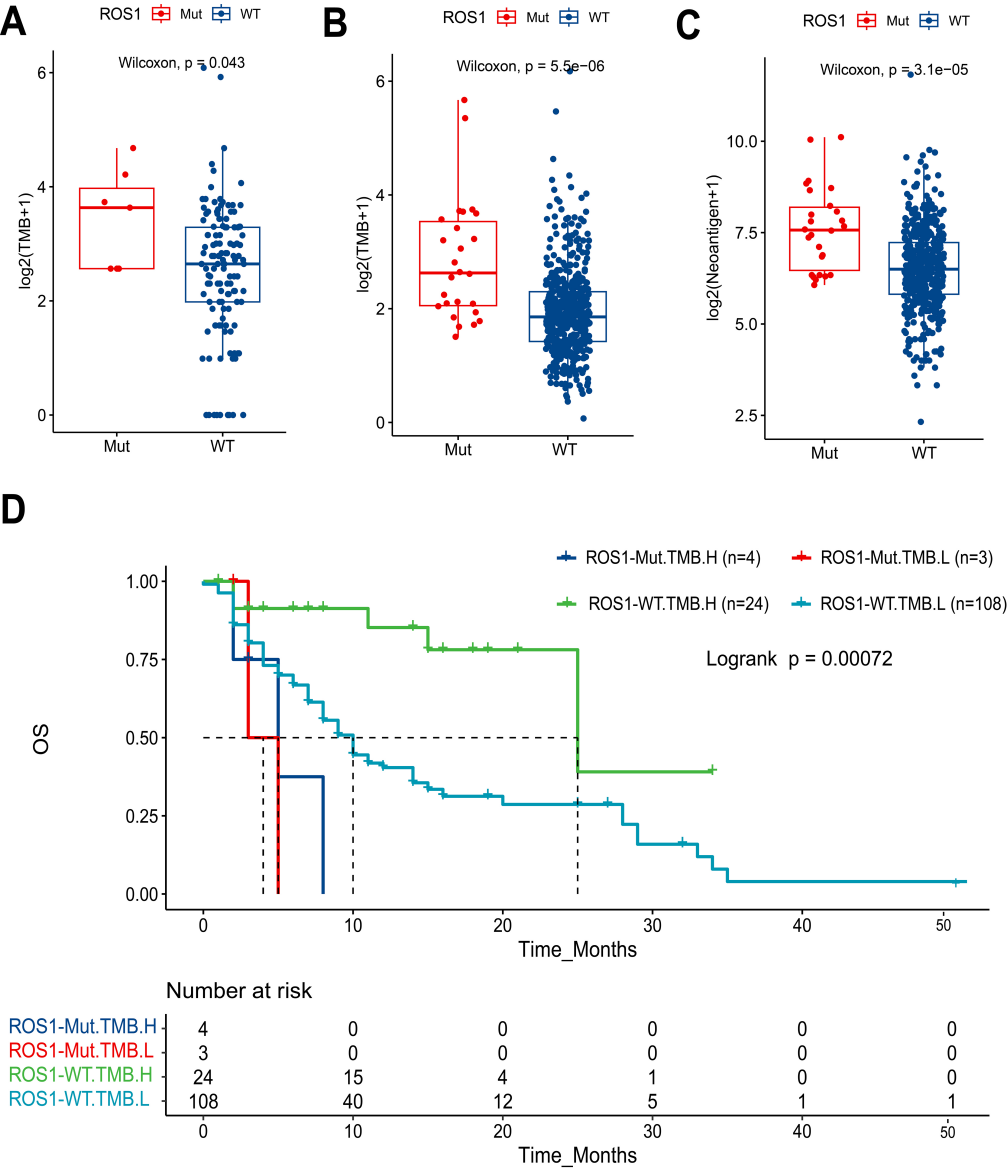
Association of *ROS1* mutations with TMB, neoantigen levels, and patient survival in HNC. Boxplots comparing log2-transformed TMB between *ROS1*-Mut and *ROS1*-WT tumors in (A) MSKCC HNC cohort and (B) TCGA HNC cohort; (C) Boxplot comparing log2-transformed neoantigen levels in TCGA HNC tumors by *ROS1* mutation status; (D) Kaplan-Meier analysis of OS stratified by *ROS1* mutation status and TMB levels. TMB: Tumor mutational burden; HNC: head and neck cancer; *ROS1*-Mut: *ROS1* mutations; *ROS1*-WT: *ROS1*-wild-type; TCGA: The Cancer Genome Atlas; OS: overall survival.

*ROS1*-Mut tumors exhibited significantly higher TMB compared to *ROS1*-WT tumors (*P* = 0.043) [Figure 3A]. This pattern was more pronounced in the TCGA HNC cohort [Figure 3B], where *ROS1*-Mut tumors showed markedly elevated TMB (*P* < 0.001). Similarly, analysis of TCGA data [Figure 3C] demonstrated that *ROS1*-Mut tumors were associated with higher neoantigen levels (*P* < 0.001), suggesting enhanced immunogenicity in ROS1-mutated cases. Despite the presumed predictive value of high TMB for ICI response, survival analysis demonstrated that *ROS1*-Mut cases with TMB-H had shorter OS compared to *ROS1*-WT cases [Figure 3D]. These findings highlight a critical interaction between *ROS1* mutations and TMB, where mutational burden fails to mitigate the aggressive biology of *ROS1*-mutant tumors in the context of ICI therapy.

### *ROS1* mutation is not a prognostic factor in HNC

To evaluate the prognostic value of *ROS1* mutation in HNC, survival analyses were conducted using TCGA data. No significant differences in progression-free survival (PFS) or OS were observed between *ROS1*-Mut and *ROS1*-WT patients (log-rank *P* = 0.26 for OS; Supplementary Figure 1), indicating that *ROS1* mutations do not serve as a prognostic factor in untreated HNC.

### *ROS1*-Mut tumors exhibit a cold immune microenvironment in HNC

**Figure 4:**
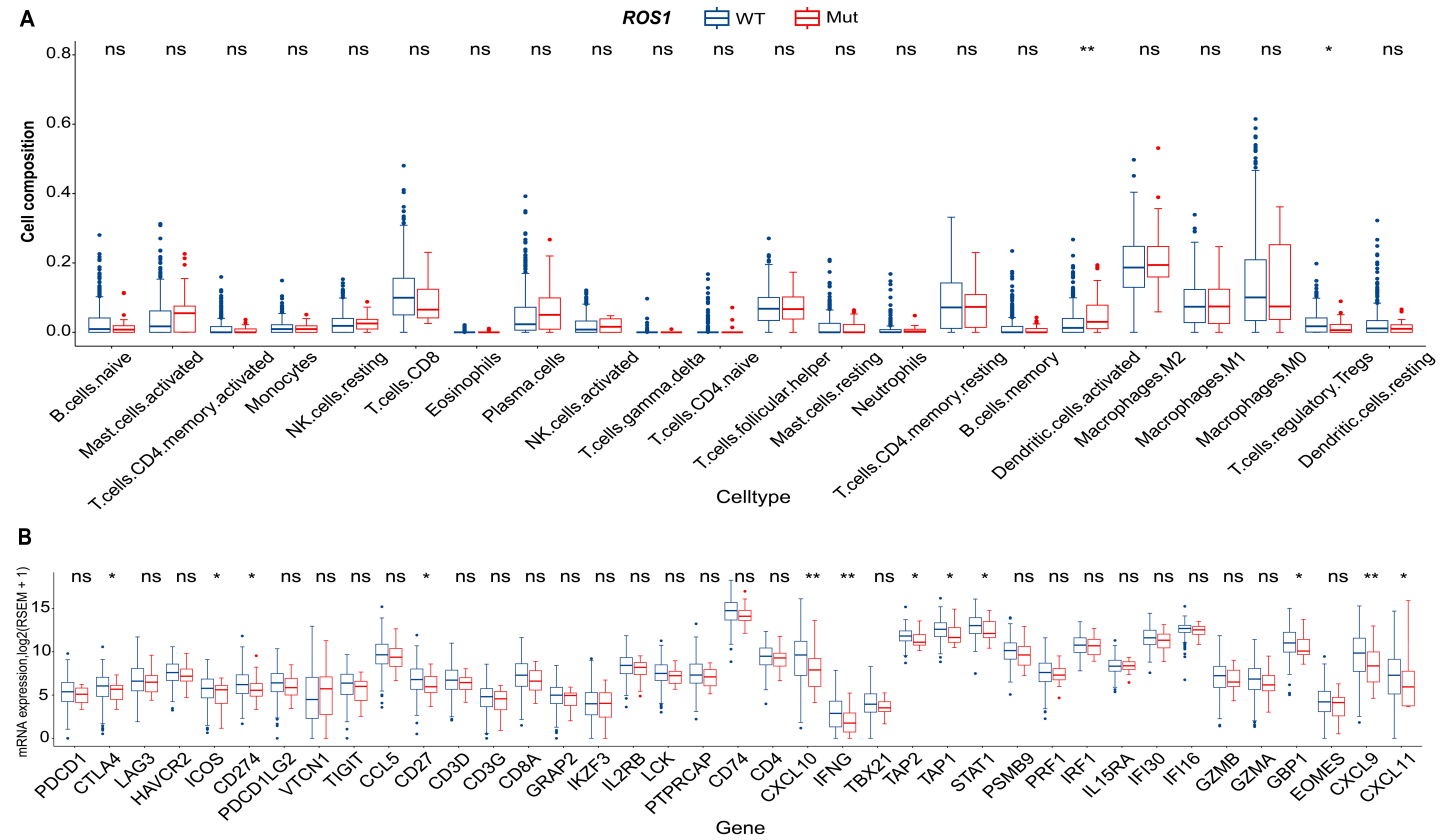
Molecular differences in immune cell composition and immune-related gene expression between *ROS1*-WT and *ROS1*-Mut tumors in the TCGA HNC cohort. (A) Comparison of 22 predefined immune cell types; (B) expression of 39 immune-related genes. ^*^P < 0.05; ^**^P < 0.01; ns: not significant (*P* ≥ 0.05). *ROS1*-WT: *ROS1*-wild-type; *ROS1*-Mut: *ROS1* mutations; TCGA: The Cancer Genome Atlas; HNC: head and neck cancer.

To investigate the molecular mechanisms underlying *ROS1*-mediated resistance to ICI therapy in HNC, RNA-seq and WES data from the TCGA-HNC cohort were analyzed. Assessment of immune cell composition revealed minimal differences between *ROS1*-Mut and *ROS1*-WT tumors [Figure 4A]. Among 22 immune cell types evaluated, only activated dendritic cells exhibited significantly higher infiltration in *ROS1*-Mut tumors (*P* < 0.01), whereas regulatory T cells (Tregs) were reduced in *ROS1*-Mut tumors (*P* < 0.05). No other immune cell types, including CD8^+^ T cells or NK cells, showed statistically significant differences. Differential expression analysis showed broad downregulation of immune-related genes in *ROS1*-Mut tumors. Notably, immune checkpoint genes (*CTLA4*, *ICOS*, *CD274*/*PD-L1*), tumor necrosis factor receptor family member *CD27*, IFN-γ mediating signaling pathway genes (*CXCL9*, *CXCL10*, *CXCL11*, *GBP1*, *IFNG*, *STAT1*), and antigen-processing genes (*TAP1*, *TAP2*) were significantly reduced in *ROS1*-Mut tumors [Figure 4B]. These results suggest that *ROS1* mutations in HNC are associated with an immunosuppressive microenvironment characterized by downregulation of immune-related genes and reduced Treg infiltration, which may contribute to resistance to ICI therapy.

### Pathway analysis links *ROS1* mutation to immune evasion mechanisms

**Figure 5:**
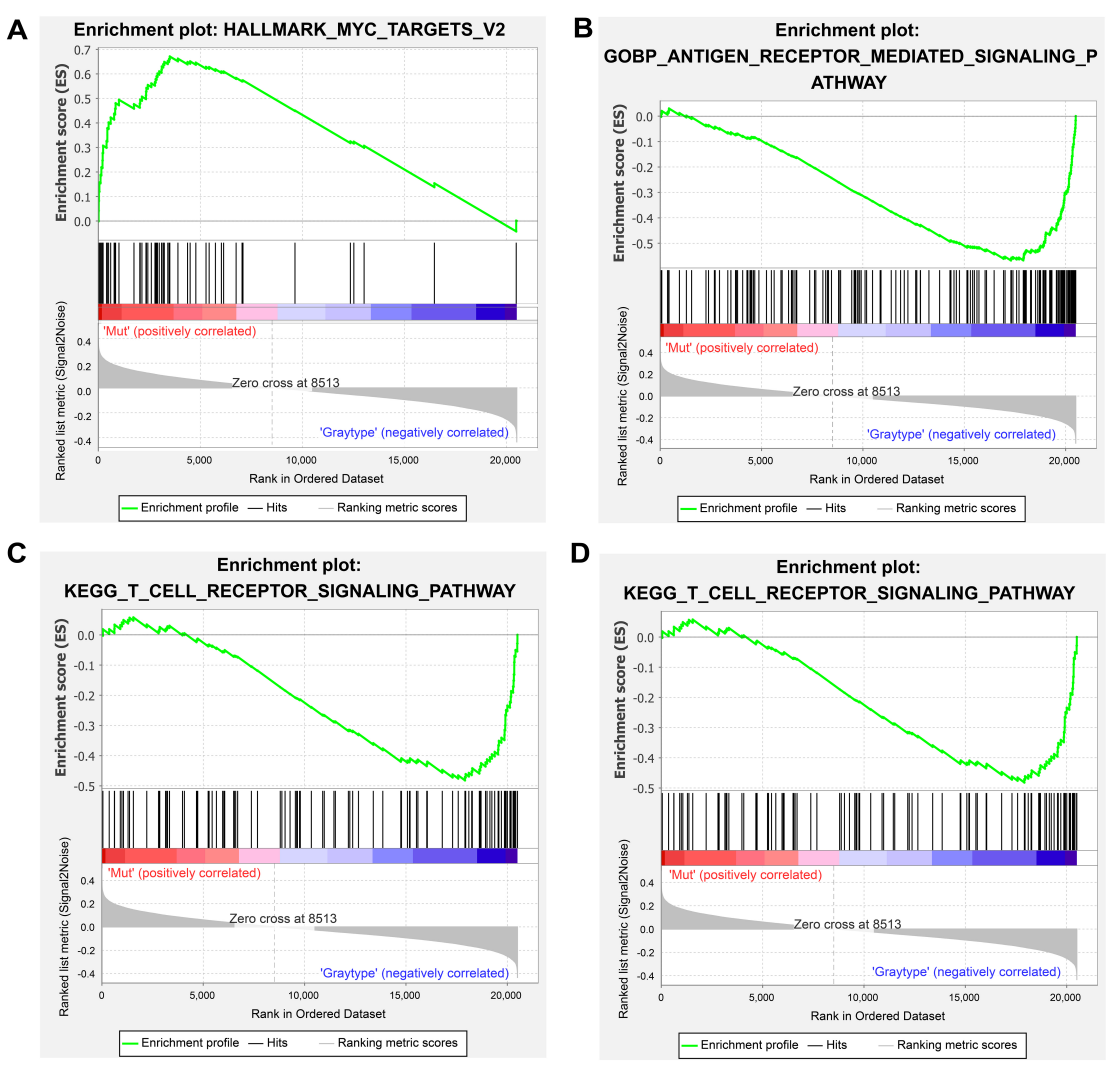
GSEA results for hallmark gene sets (A) and KEGG pathways (B-D). A *t*-value > 0 (red) indicates higher scores in *ROS1*-Mut tumors; *t*-value < 0 (blue) indicates higher scores in *ROS1*-WT tumors. GSEA: Gene set enrichment analysis; *ROS1*-Mut: *ROS1* mutations; *ROS1*-WT: *ROS1*-wild-type.

GSEA revealed distinct pathway activation patterns between *ROS1*-Mut and *ROS1*-WT tumors, elucidating potential mechanisms of ICI resistance. In *ROS1*-Mut tumors, genes associated with the *MYC* signaling pathway were significantly upregulated [Figure 5A], mediating tumor proliferation, metabolic adaptation, and immune evasion. In contrast, immune-activating pathways including antigen receptor-mediated signaling, T cell receptor (TCR) signaling, and cell adhesion molecules were downregulated in *ROS1*-Mut tumors [Figure 5B-D]. These findings collectively highlight that *ROS1* mutations drive transcriptional reprogramming toward an immunosuppressive phenotype.

**Figure 6:**
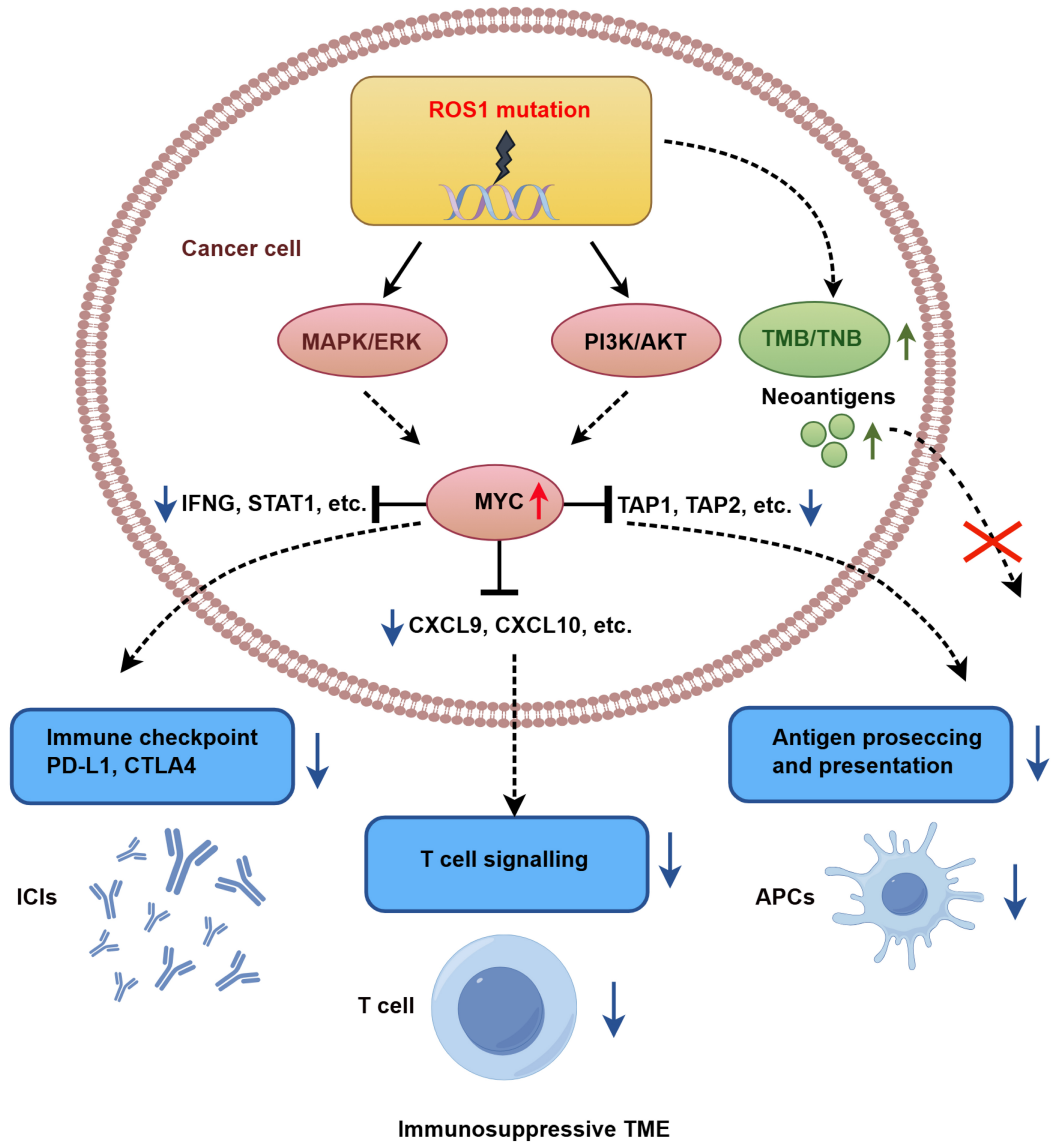
Proposed mechanism of *ROS1* mutation-driven immune evasion in HNC. HNC: Head and neck cancer; APCs: antigen-presenting cells; ICIs: immune checkpoint inhibitors; TMB: tumor mutational burden; TME: tumor microenvironment; TNB: tumor neoantigen burden.

Integrative multi-omics analyses further revealed that *ROS1*-mutant tumors exhibit hyperactivation of the *MYC* pathway, which transcriptionally represses IFN-γ signature genes (e.g., *IFNG*, *STAT1*), T cell chemokines (e.g., *CXCL9*, *CXCL10*), and components of the antigen presentation machinery (*TAP1* and *TAP2*), collectively fostering an immunosuppressive TME. Despite elevated TMB and neoantigen load, these tumors show impaired CD8^+^ T cell infiltration and reduced responsiveness to ICI therapy. Figure 6 provides a schematic overview of the proposed mechanistic cascade by which *ROS1* mutations drive immune evasion and ICI resistance in HNC.

## DISCUSSION

This study demonstrates that *ROS1*-Mut tumors develop an immunosuppressive TME, characterized by diminished CD8^+^ T cell infiltration, downregulation of key immune-related genes (e.g., *CXCL9*/*10*, *IFNG*, *PD-L1*), and impaired interferon-γ (IFN-γ) signaling. These findings identify *ROS1* mutation as a potential predictive marker of resistance to ICIs in HNC, although clinical validation in larger cohorts is needed.

Despite elevated TMB and neoantigen load in *ROS1*-Mut tumors - features typically associated with enhanced ICI response - these patients exhibited significantly shorter OS (median OS: 5.0 *vs.* 11.0 months, HR = 3.22, *P* = 0.011). The paradoxical coexistence of high TMB with poor ICI response highlights the limitations of mutational burden as a universal biomarker. While TMB generally correlates with neoantigen visibility, *ROS1*-Mut tumors may override this immunogenic advantage through *MYC*-mediated transcriptional reprogramming. This is supported by significant downregulation of antigen presentation machinery (*TAP1*/*TAP2*) and T cell chemotaxis genes (*CXCL9*/*CXCL10*) in our transcriptomic data [Figure 4B], consistent with established MYC-immune suppression mechanism ^[27-32]^.

Our GSEA results [Figure 5A], together with the observed downregulation of MHC-I pathway genes [Figure 4B], suggest that *MYC* activation contributes to an immunosuppressive TME where a high neoantigen load becomes biologically irrelevant, mirroring *MYC*-driven immune evasion reported in pancreatic ductal adenocarcinoma (PDAC)^[31]^ and hepatocellular carcinoma (HCC)^[32]^. Similar phenomena have been observed in other oncogenic alterations, such as *MDM2*/*4* amplification, where ICI-induced hyperprogression correlates with suppressed T cell infiltration^[27]^. *JAK1/2* mutations have also been associated with accelerated tumor growth post-ICI due to defective MHC-I expression and resistance to T cell cytotoxicity in melanoma^[28]^.

The upregulation of *MYC* signaling in *ROS1*-Mut tumors aligns with prior studies linking MYC to immune suppression via downregulation of MHC class I molecules and recruitment of immunosuppressive myeloid cells^[29-32]^. These defects in antigen presentation represent a rate-limiting step that impedes neoantigen visibility^[33]^, explaining the discordance between high TMB and poor ICI response in *ROS1*-mutant tumors. Concurrently, suppression of IFN-γ response genes (*CXCL9*, *CXCL10*, *STAT1*) mirrors observations in melanoma and lung cancer, where similar transcriptional silencing correlates with T cell exclusion and ICI resistance^[34,35]^. Additionally, the reduced expression of *PD-L1* (*CD274*) and *CTLA4* in *ROS1*-Mut tumors might further impair ICI efficacy. Collectively, these findings highlight that checkpoint molecule expression or TMB alone may be insufficient to predict ICI response, emphasizing the need for composite biomarkers that integrate genomic and immune profiling.

Clinically, *ROS1* mutation status may serve as a stratification tool to identify HNC patients unlikely to benefit from ICIs. This context-dependent predictive role parallels findings for *TGFBR2* mutations in NSCLC^[36]^ and *TP53* mutations in melanoma^[37]^, but contrasts with *DYNC2H1* mutations, which enhance immunogenicity^[38]^. The variable effects of kinase mutations highlight the need for biomarker-driven therapeutic strategies.

Therapeutically, *ROS1*-Mut HNC may benefit from combinatorial strategies targeting both immune evasion pathways and oncogenic drivers. Preclinical studies show that ROS1 inhibitors (e.g., entrectinib) can reverse oncogenic signaling^[13,26]^, while MYC inhibition can restore antigen presentation in other models^[39]^. Thus, combinatorial strategies represent a biologically grounded approach warranting further evaluation in *ROS1*-mutant systems.

This study has limitations. *ROS1* mutations occur at relatively low incidence in HNC (5%-7%), though this subgroup exhibits significant clinical detriment (median OS: 5 months). Our biomarker claims are constrained by the small sample size of *ROS1*-mutant cases (*n* = 7 in the ICI-treated cohort), heterogeneity of variants (predominantly VUS), and lack of functional validation. While statistically significant, these findings require prospective validation in larger cohorts and mechanistic studies to confirm *ROS1*’s role in immune evasion. Additionally, the absence of prognostic significance in TCGA (non-ICI-treated cohort) underscores its context-specific predictive role, akin to *KRAS* mutations in colorectal cancer^[40]^.

In conclusion, *ROS1*-mutant HNC defines a molecular subset with intrinsic ICI resistance driven by MYC-mediated immunosuppression. If validated prospectively, integrating *ROS1* status with TMB and PD-L1 expression could optimize patient stratification for ICI therapy, though further studies are essential to establish clinical utility.

## DECLARATIONS

### Availability of data and materials

The datasets used and/or analyzed during the current study are available from the corresponding authors upon reasonable request.

### Copyright

© The Author(s) 2025.

## References

[1] Bray F, Laversanne M, Sung H (2024). Global cancer statistics 2022: GLOBOCAN estimates of incidence and mortality worldwide for 36 cancers in 185 countries. CA Cancer J Clin.

[2] Petrelli F, Lorini L, Paderno A (2025). Treatment of primary tumor in metastatic head and neck carcinoma: a systematic review and meta-analysis. Oral Oncol.

[3] Szturz P, Fuereder T, Guo Y (2025). Treatment decision-making factors and sequencing in recurrent and/or metastatic squamous cell carcinoma of the head and neck. Cancer Treat Rev.

[4] Liu X, Harbison RA, Varvares MA, Puram SV, Peng G (2025). Immunotherapeutic strategies in head and neck cancer: challenges and opportunities. J Clin Invest.

[5] Ferris RL, Blumenschein G Jr, Fayette J (2016). Nivolumab for recurrent squamous-cell carcinoma of the head and neck. N Engl J Med.

[6] Seiwert TY, Burtness B, Mehra R (2016). Safety and clinical activity of pembrolizumab for treatment of recurrent or metastatic squamous cell carcinoma of the head and neck (KEYNOTE-012): an open-label, multicentre, phase 1b trial. Lancet Oncol.

[8] Johnson DE, Burtness B, Leemans CR, Lui VWY, Bauman JE, Grandis JR (2020). Head and neck squamous cell carcinoma. Nat Rev Dis Primers.

[9] Samstein RM, Lee CH, Shoushtari AN (2019). Tumor mutational load predicts survival after immunotherapy across multiple cancer types. Nat Genet.

[10] Sharma P, Allison JP (2015). The future of immune checkpoint therapy. Science.

[11] Rizvi NA, Hellmann MD, Snyder A (2015). Cancer immunology. Mutational landscape determines sensitivity to PD-1 blockade in non-small cell lung cancer. Science.

[12] Snyder A, Makarov V, Merghoub T (2014). Genetic basis for clinical response to CTLA-4 blockade in melanoma. N Engl J Med.

[14] Waliany S, Lin JJ (2024). Taletrectinib: TRUST in the continued evolution of treatments for ROS1 fusion-positive lung cancer. J Clin Oncol.

[15] Li W, Xiong A, Yang N (2024). Efficacy and safety of taletrectinib in Chinese patients with *ROS1+* non–small cell lung cancer: the Phase II TRUST-I study. J Clin Oncol.

[16] Canon J, Rex K, Saiki AY (2019). The clinical KRAS(G12C) inhibitor AMG 510 drives anti-tumour immunity. Nature.

[17] Gainor JF, Shaw AT, Sequist LV (2016). EGFR mutations and ALK rearrangements are associated with low response rates to PD-1 pathway blockade in non-small cell lung cancer: a retrospective analysis. Clin Cancer Res.

[18] Ambrosini M, Rousseau B, Manca P (2024). Immune checkpoint inhibitors for POLE or POLD1 proofreading-deficient metastatic colorectal cancer. Ann Oncol.

[20] Angelova M, Charoentong P, Hackl H (2015). Characterization of the immunophenotypes and antigenomes of colorectal cancers reveals distinct tumor escape mechanisms and novel targets for immunotherapy. Genome Biol.

[21] Nielsen M, Lundegaard C, Blicher T (2007). NetMHCpan, a method for quantitative predictions of peptide binding to any HLA-A and -B locus protein of known sequence. PLoS One.

[23] Love MI, Huber W, Anders S (2014). Moderated estimation of fold change and dispersion for RNA-seq data with DESeq2. Genome Biol.

[24] Danaher P, Warren S, Dennis L (2017). Gene expression markers of tumor infiltrating leukocytes. J Immunother Cancer.

[25] Yu G, Wang LG, Han Y, He QY (2012). clusterProfiler: an R package for comparing biological themes among gene clusters. OMICS.

[26] Drilon A, Jenkins C, Iyer S, Schoenfeld A, Keddy C, Davare MA (2021). ROS1-dependent cancers - biology, diagnostics and therapeutics. Nat Rev Clin Oncol.

[27] Kato S, Goodman A, Walavalkar V, Barkauskas DA, Sharabi A, Kurzrock R (2017). Hyperprogressors after immunotherapy: analysis of genomic alterations associated with accelerated growth rate. Clin Cancer Res.

[28] Zaretsky JM, Garcia-Diaz A, Shin DS (2016). Mutations associated with acquired resistance to PD-1 blockade in melanoma. N Engl J Med.

[29] Casey SC, Tong L, Li Y (2016). MYC regulates the antitumor immune response through CD47 and PD-L1. Science.

[30] Fan Y, Song S, Li Y (2023). Galectin-3 cooperates with CD47 to suppress phagocytosis and T-cell immunity in gastric cancer peritoneal metastases. Cancer Res.

[31] Krenz B, Gebhardt-Wolf A, Ade CP (2021). MYC- and MIZ1-dependent vesicular transport of double-strand RNA controls immune evasion in pancreatic ductal adenocarcinoma. Cancer Res.

[32] Dhanasekaran R, Hansen AS, Park J (2023). MYC overexpression drives immune evasion in hepatocellular carcinoma that is reversible through restoration of proinflammatory macrophages. Cancer Res.

[34] Spranger S, Bao R, Gajewski TF (2015). Melanoma-intrinsic β-catenin signalling prevents anti-tumour immunity. Nature.

[35] Gao J, Shi LZ, Zhao H (2016). Loss of IFN-γ pathway genes in tumor cells as a mechanism of resistance to anti-CTLA-4 therapy. Cell.

[36] Li T, Wang H, Xu J (2021). TGFBR2 mutation predicts resistance to immune checkpoint inhibitors in patients with non-small cell lung cancer. Ther Adv Med Oncol.

[37] Xiao W, Du N, Huang T (2018). TP53 mutation as potential negative predictor for response of anti-CTLA-4 therapy in metastatic melanoma. EBioMedicine.

[38] Yang L, Feng Y, Liu X (2025). DYNC2H1 mutation as a potential predictive biomarker for immune checkpoint inhibitor efficacy in NSCLC and melanoma. Invest New Drugs.

[39] Kortlever RM, Sodir NM, Wilson CH (2017). Myc cooperates with Ras by programming inflammation and immune suppression. Cell.

[40] Dienstmann R, Vermeulen L, Guinney J, Kopetz S, Tejpar S, Tabernero J (2017). Consensus molecular subtypes and the evolution of precision medicine in colorectal cancer. Nat Rev Cancer.

