# Supplementary material for: ROS1 mutations promote an immunosuppressive tumor microenvironment via MYC to confer immune evasion in head and neck cancer
Source: Cancer Drug Resist. 2025 Aug 22;8:42. doi: 10.20517/cdr.2025.124 (PMC12378350; doi:10.20517/cdr.2025.124)
Supplement: Supplementary file 1 [file cdr-8-42-SupplementaryMaterials.zip › cdr80124-Supplementary Figure 1.docx]

**Supplementary Materials**

***ROS1* mutations promote an immunosuppressive tumor microenvironment via *MYC* to confer immune evasion in head and neck cancer**

**Chao Fang^1,2,#^, Qin Zhang^3,#^, Rui Fang^1,2,#^, Ying Li^1,2^, Jing Bai^1,2^, Xiaojing Huang^1^, Jingting Lu^1^,** **Dongsheng Chen^3^, Yanxiang Zhang^3^, Zuhong Chen^1,2^**

^1^The School of Clinical Medicine, Fujian Medical University, Fuzhou 350000, Fujian, China.

^2^Department of Otolaryngology-Head & Neck Surgery, The First Hospital of Putian, Putian 351100, Fujian, China.

^3^State Key Laboratory of Neurology and Oncology Drug Development, Jiangsu Simcere Diagnostics Co., Ltd., Nanjing 210018, Jiangsu, China.‌

^#^Authors contributed equally.

**Correspondence to:** Prof. Zuhong Chen, The School of Clinical Medicine, Fujian Medical University, No. 88 Jiaotong Road, Fuzhou 350000, Fujian, China. E-mail: [zuhongchen@126.com](mailto:zuhongchen@126.com) (lead contact); Dr. Yanxiang Zhang, State Key Laboratory of Neurology and Oncology Drug Development, Jiangsu Simcere Diagnostics Co., Ltd., No. 699-18 Xuanwu Street, Nanjing 210018, Jiangsu, China. E-mail: [yxzhang2008@hotmail.com](mailto:yxzhang2008@hotmail.com)

**
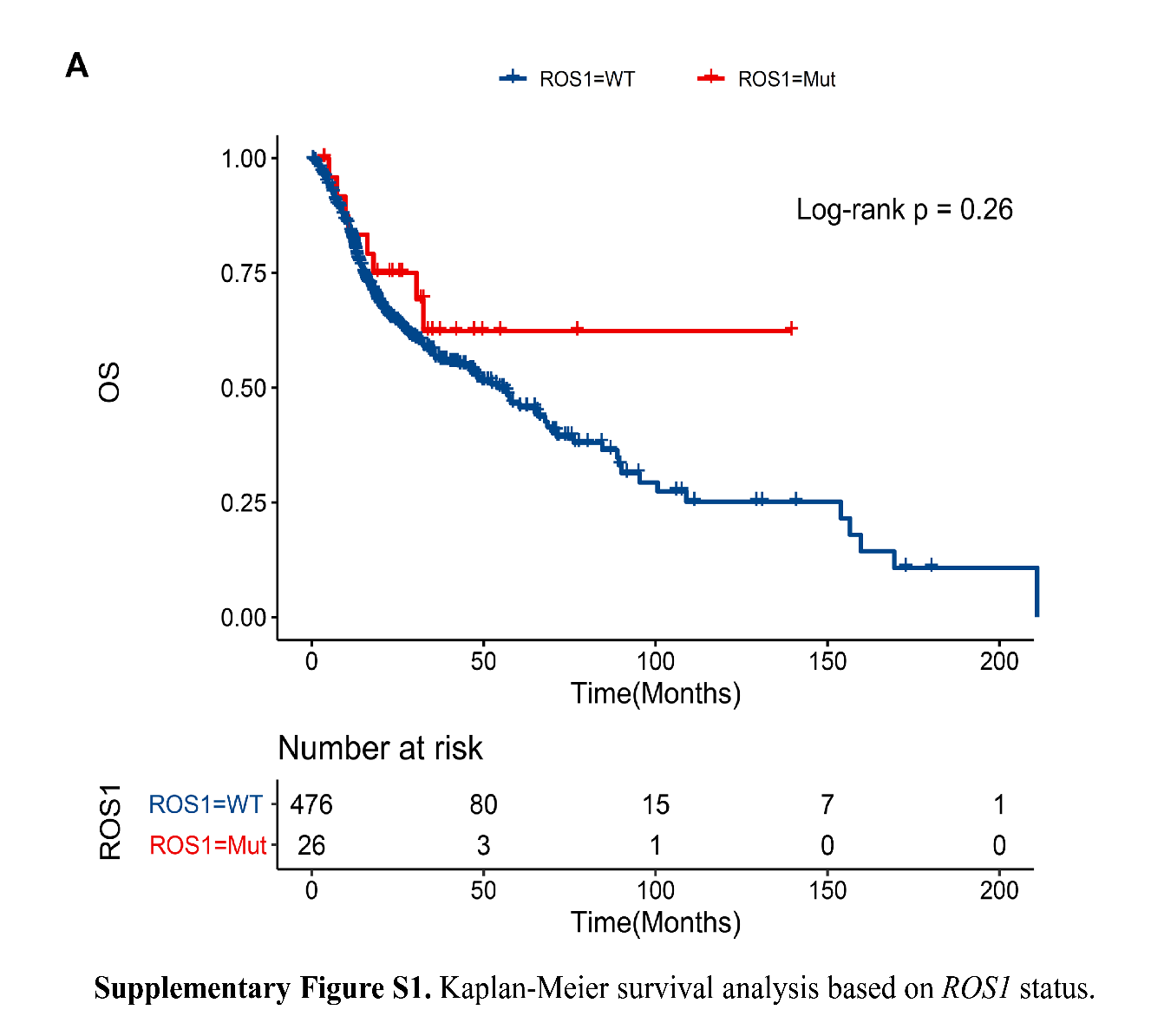
**

**Supplementary Figure 1.** Kaplan-Meier survival analysis based on *ROS1* status.
